# Attolitre-sized lipid bilayer chamber array for rapid detection of single transporters

**DOI:** 10.1038/srep11025

**Published:** 2015-06-08

**Authors:** Naoki Soga, Rikiya Watanabe, Hiroyuki Noji

**Affiliations:** 1 Department of Applied Chemistry, Graduate School of Engineering, The University of Tokyo, Bunkyo-ku, Tokyo 113-8656, Japan; 2 PRESTO, Japan Science and Technology, Bunkyo-ku, Tokyo 113-8656, Japan; 3 CREST, Japan Science and Technology Agency, Chiyoda-ku, Tokyo 102-0075, Japan

## Abstract

We present an attolitre-sized arrayed lipid bilayer chamber system (aL-ALBiC) for rapid and massively parallel single-molecule assay of membrane transporter activity. Because of the small reaction volume (200 aL), the aL-ALBiC performed fast detection of single transporter activity, thereby enhancing the sensitivity, throughput, and accuracy of the analysis. Thus, aL-ALBiC broadens the opportunities for single-molecule analysis of various membrane transporters and can be used in pharmaceutical applications such as drug screening.

Membrane transporters play pivotal roles in controlling the uptake and efflux of ionic species, entry of nutrients, regulation of metabolite concentrations, or expulsion of drugs and other toxic substance^1^,^2^. Transporters, *e.g*. multidrug transporters, have frequently been the targets of pharmaceutical research because of their physiological importance^3^,^4^,^5^. Several massively parallel and highly quantitative systems for analysis of transporters have been developed^6^ to contribute to high-throughput drug discovery. One of the most robust systems for membrane transporter analysis is patch clamp recording, which measures the flux of a charged substrate as an electric current under constant electrical voltage across a membrane. Recent developments have automated patch clamp recordings, thus enabling massive and parallel analysis of transporter activities, *e.g*. ion channels^7^,^8^; however, most transporters cannot generate sufficient electric current (>10^7^ molecules s^−1^) for detection due to low transport rates (<10^2^ molecules s^−1^). Moreover, patch clamp recording cannot detect the flux of electrically neutral substrates. Therefore, the development of a more versatile system is desired.

A microsystem for arrayed micro-sized reactors sealed with lipid bilayers is another option for membrane transporter analysis; in these systems, transport activity is measured optically based on substrate accumulation or consumption in the chamber^6^,^9^,^10^,^11^,^12^,^13^,^14^. The microsystems enhance the sensitivity and throughput of transporter analysis; however, it remains difficult to measure transport activity of single molecules due to large reactor volumes or low lipid bilayer formation efficiency^15^. To address this issue, we recently developed the arrayed lipid bilayer chamber system (ALBiC)^16^,^17^, which contains more than 10,000 arrayed femtolitre chambers (7 fL) sealed with stable 4-μm lipid bilayers. The ALBiC system improved the efficiency of lipid bilayer formation to more than 90% and performed highly sensitive and parallel analysis of membrane transport, which enables single molecule analysis of extremely low transport activities (<10 molecules s^−1^). Although it is powerful for highly sensitive and quantitative analysis, detection of slow transport activity requires a relatively long time (>2000 s) because more than 10,000 transport substrates must accumulate in the chamber (μM concentrations) to be detected by fluorescent indicators, and the transport rate of most transporters is less than 100 s^−1^. Therefore, rapid detection of slow transport activity requires a much smaller chamber volume than reported previously^16^,^17^. In this study, we address this issue by developing a novel ALBiC system with attolitre reaction chambers (aL-ALBiC) to achieve faster single-molecule detection of transporter activity in a highly sensitive and quantitative manner.

## Results

### Formation of lipid bilayer chambers.

**Figure 1:**
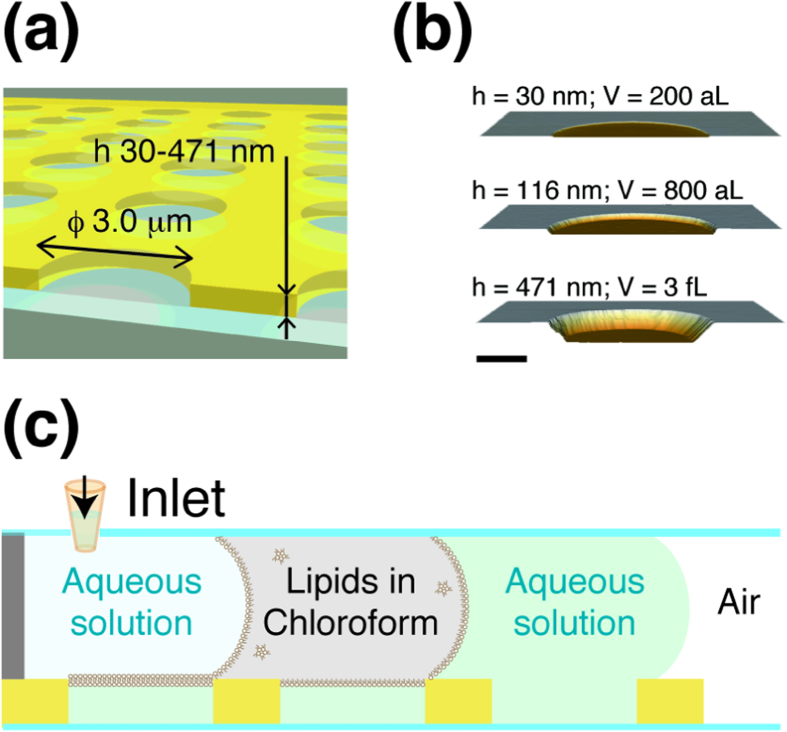
Arrayed lipid bilayer chamber system (ALBiC). (**a**) Schematic image of fabricated nano-device containing ~10,000 arrays of hydrophilic/hydrophobic structures (diameter, 3.0 μm; height, 30–471 nm). (**b**) Atomic force microscopy (AFM) image of three different fabricated structures. The heights of the chambers are 30 ± 5 nm, 116 ± 13 nm, and 471 ± 42 nm as measured by 3D laser scanning microscopy, atomic force microscopy, and transmission electron microscopy. The scale bar represents 1 μm. (**c**) Schematic of the process for formation of lipid bilayer membranes on the nano-device.

**Figure 2:**
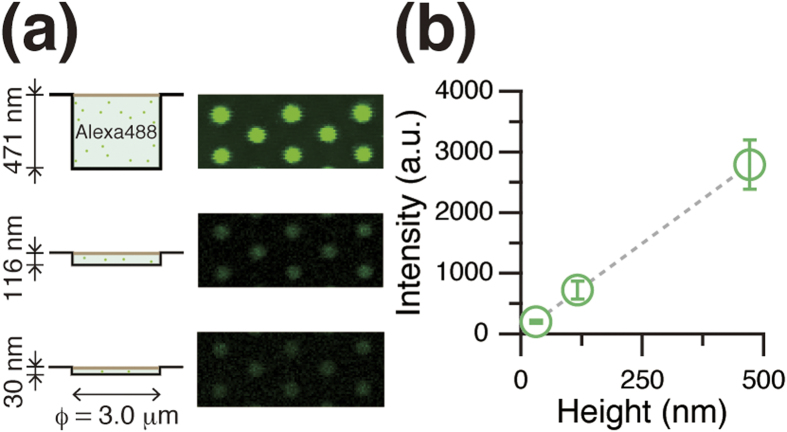
Height dependence of encapsulated dye in ALBiCs. (**a**) Schematic (left) and fluorescent image (right) of ALBiCs. Alexa 488 fluorescent dye (5 μM) was encapsulated in chambers with three different heights. (**b**) Height dependence of fluorescence intensity of 5 μM Alexa 488 encapsulated into chambers. The grey dashed line represents a linear fit with a correlation coefficient of 1.0.

Using conventional photolithography, we fabricated nano-devices with more than 10,000 through-hole structures (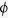 = 3.0 μm) on a hydrophobic fluorine polymer on a hydrophilic glass substrate (Fig. 1a)^16^,^17^,^18^,^19^. To examine the feasibility of aL-ALBiC, we prepared three types of chambers with the same orifice size (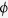 = 3.0 μm) and different heights, *i.e*. 471 nm, 116 nm, and 30 nm, corresponding to 3.3 fL, 800 aL, and 200 aL volumes, respectively (Fig. 1b). The chamber orifice size, which defines the exposed surface area of the lipid membrane, contributes markedly to the incorporation efficiency of membrane transporters as previously reported^12^, so we used a consistent orifice size to ensure equal incorporation efficiency in the transporter assay. To exchange solutions on the device, the flow cell was constructed by assembling the fabricated device with a spacer sheet and CYTOP-coated glass cover, which has an access port for injection of solution. Lipid bilayers were formed on the orifice of each chamber after sequential injection of aqueous solution and organic solvent containing lipids as previously reported (Fig. 1c). First, an aqueous solution containing Alexa 488 fluorescent dye was infused in the flow cell. Then, we infused 2 mg·mL^−1^ DOPE/DOPG (1:1 [w:w]) in chloroform to flush out the first aqueous solution. Water-in-solvent droplets closed with lipid monolayers were formed in the individual chambers. Finally, a second aqueous solution was infused to remove the lipid solution. The residual lipids formed lipid bilayers on the orifice of each chamber with 99.3% efficiency for the 3.3-fL chambers (13796 sealed out of 13890 chambers), 96.3% efficiency for the 800-aL chambers (15105 sealed out of 15682 chambers), and 72.1% efficiency for 200-aL chambers (9537 sealed out of 13280 chambers) (Fig. 2a). These efficiencies were much higher than those of previously reported microsystems (~50%)^20^,^21^,^22^, and the lipid bilayers were stable for at least 2 h (Fig. S1b). Bright-field images taken after lipid membrane formation showed circular interference patterns in the individual chambers (Fig. S2a), which are characteristic optical features of thin lipid bilayers, termed the Plateau–Gibbs border^16^,^23^,^24^. Thus, we achieved highly efficient formation of stable and uniform lipid bilayers all over the device, even for the 200-aL chambers (30-nm height), where the aspect ratio of chamber height to lipid orifice size was 30 nm/3 μm = 0.01, which is much smaller than previously reported^9^,^10^,^11^,^12^,^13^,^20^. This highly efficient formation is due to the advantage of chloroform as a solvent, as we previously reported. The chloroform retained in lipid membranes can dissolve in aqueous solution since the solubility of chloroform in water is much higher than that of the other organic solvents, *e.g. n*-decane, used in previous work. Thus, the lipid membranes spontaneously form bilayers without the mechanical perturbation, *e.g*. hydraulic pressure, required for other microsystems, resulting in more efficient bilayer formation^20^,^21^,^22^.

### Confirmation of volume in lipid bilayer chambers

To determine the droplet volume in chambers encapsulated by lipid bilayers, we measured the intensity of a fixed concentration of fluorescent dye (5 μM Alexa 488) dissolved in the droplet. The fluorescence intensity is proportional to the droplet thickness since it is much smaller than the focal depth of the microscope, ~1 μm. The intensities of the pixels within a chamber were homogeneous, *i.e*. the droplet thickness was uniform throughout the chamber, indicating that the lipid bilayer was horizontally supported on the chamber orifice, and the droplet formed a cylindrical shape defined by the chamber (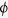 = 3.0 μm) (Fig. 2a). The fluorescence intensity of each droplet was clearly proportional to the chamber height (Fig. 2). Therefore, the droplet volume in aL-ALBiC was well defined by the chamber shape at attolitre-scale volumes.

### Fluorescence recovery after photobleaching.

The aL-ALBiC devices formed in this study were hermetically sealed for stable retention of the solute, as confirmed by fluorescence recovery after photobleaching (FRAP). The attolitre chambers were filled with 10 μM Alexa 488, which was used as a transport substrate for the α-haemolysin assay described below. Fluorescent images of Alexa 488 were recorded at 100-s intervals using a confocal microscope. Using the laser scanning system, one of the chambers was selectively photobleached (Fig. S1a). Subsequent measurements of fluorescence intensity showed that the bleached chamber did not recover its fluorescence, suggesting that the dye did not diffuse within the 2-h observation period (Fig. S1). Thus, the crosstalk between the chambers was negligible, supporting the integrity of the hermetic seal generated by the lipid membrane. This allows for quantitative analysis of membrane transporter activity.

### Fast detection of transporter activity.

**Figure 3:**
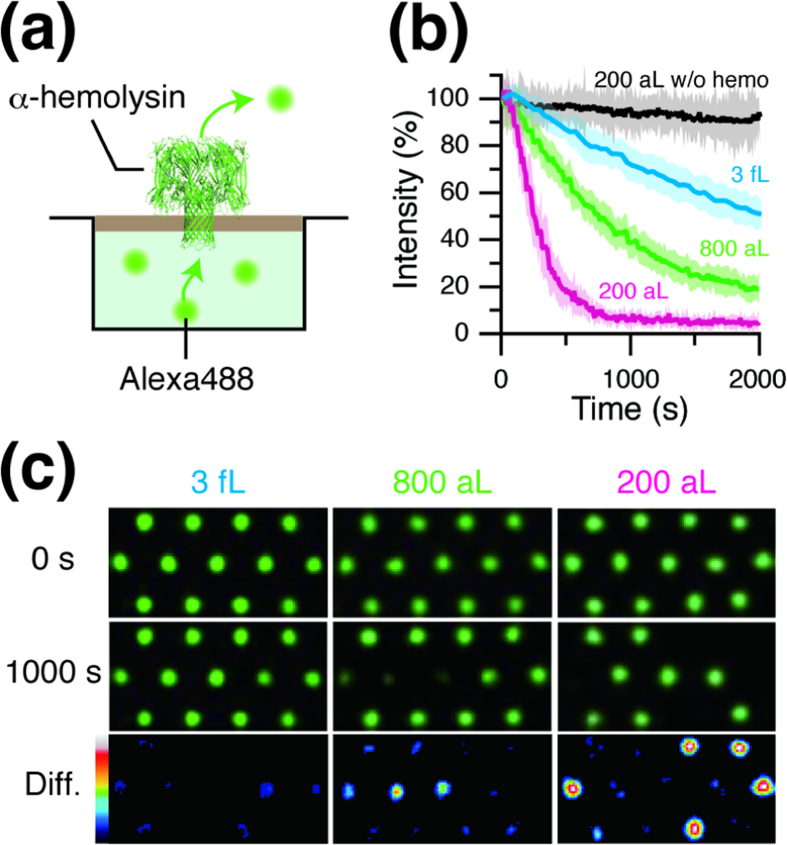
Fluorescent detection of passive transport through the α-haemolysin nanopore. (**a**) Schematic of passive transport by α-haemolysin. The encapsulated Alexa 488 passively diffuses to the outer solution via the α-haemolysin nanopore. (**b**) Time course of passive transport by 0.1 μg/mL α-haemolysin with three types of ALBiCs as indicated. Each solid line represents the average over eight or more representative chambers. Error shades in each trace represent the standard deviation. (**c**) Fluorescent image of passive transport activity with three types of ALBiCs as indicated. The images were recorded just after injection of α-haemolysin (t = 0, top) and 1,000 s later (middle). The bottoms (diff.) show the difference between each image at 0 s and 1,000 s as a color gradient.

We defined the aL-ALBiC as the micro-nano system with the smallest reactor volume (~200 aL) sealed with lipid bilayers. Due to the ultra-small reactor volume, we expected that aL-ALBiC would improve the detection sensitivity and enable faster detection of transporter activities. As a proof-of-concept, we conducted passive transport assays of α-haemolysin using aL-ALBiC. α-haemolysin, categorized as a transporter protein^2^,^25^, is a transmembrane pore-forming toxin (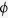 = ~1 nm) that has been widely used as a model transporter protein (Fig. 3a)^6^,^10^,^11^,^12^,^13^,^14^,^16^,^26^,^27^. We previously determined the simple physicochemical model for the passive transport activity of α-haemolysin in ALBiC^16^, where diffusion of the dye molecules from the chamber through an α-haemolysin nanopore is the kinetic bottleneck and obeys Fick’s law. In this model, the fluorescence intensity, *F*(t), of the transport substrate, Alexa 488, encapsulated in the chamber is expressed by equation (1):


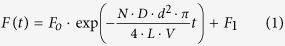


where *N* is the number of α-haemolysin pores reconstituted in the lipid membrane, *D* is the diffusion coefficient of Alexa 488, *d* is the diameter of the α-haemolysin pore (~1 nm), *L* is the length of the α-haemolysin pore (~10 nm), and *V* is the volume of the chamber^16^. According to equation (1), the fluorescence intensity of the chamber exponentially decreases due to the passive transport activity of α-haemolysin with a rate constant inversely proportional to the volume of the chamber. Thus, we expected that the detection time for transport activities (the time constant of fluorescent decay), would become shorter in proportion to the chamber volume.

**Figure 4:**
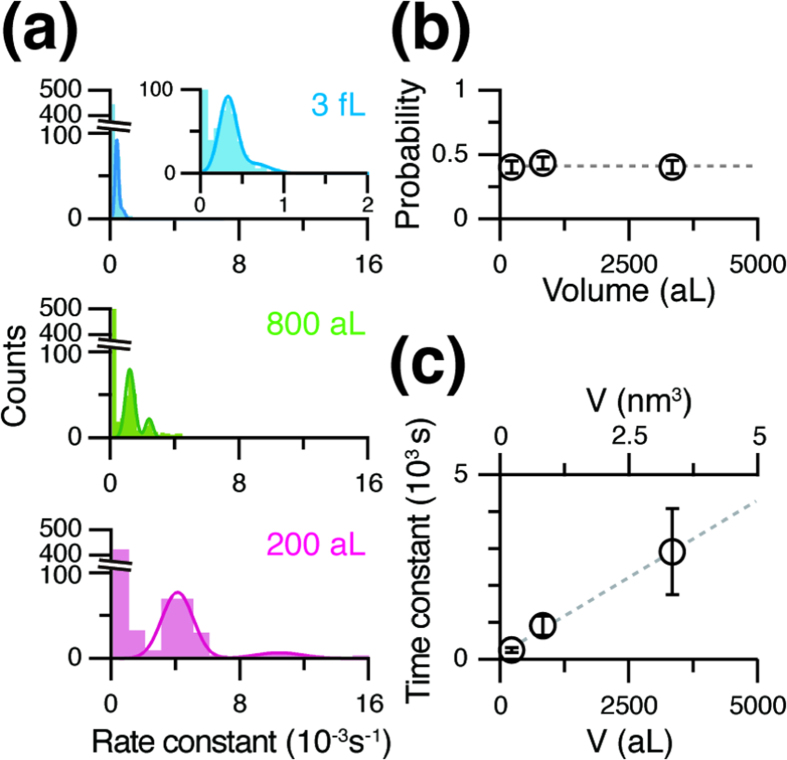
Volume dependence of rate constant in passive transport. (**a**) Histogram of rate constants for passive transport obtained from single-exponential fits to the traces in Fig. 3b. Each bin is 0.00007 s^−1^ in 3-fL chambers (black, top), 0.00025 s^−1^ in 800-aL chambers (cyan, middle), and 0.001 s^−1^ in 200-aL chambers (magenta, bottom). The dark-coloured lines show double Gaussian fits to each histogram excluding the left-most, which corresponds to empty chambers. The inset shows a magnified region between 0–100 counts on the *y*-axis. The rate constants of passive transport by single α-haemolysin molecules obtained from the Gaussian fits are 0.26 × 10^−3^, 1.1 × 10^−3^, and 4.0 × 10^−3^ s^−1^ for chambers with 471 nm, 116 nm, and 30 nm heights, respectively. (**b**) The proportion of active chambers reflecting α-haemolysin incorporation. The data and errors are obtained from Fig. 4a. The grey dashed line represents the average of all probabilities, 41.3%. (**c**) Volume dependence of the rate constant for fluorescent decay by single α-haemolysin. The data and errors are obtained from Fig. 4a. The grey dashed line represents a linear fit with a correlation coefficient of 1.0.

We measured the passive transport activity of α-haemolysin using aL-ALBiC with different chamber heights. A fluorescent dye (10 μM Alexa 488) was encapsulated in the chambers as a transport substrate before injecting α-haemolysin. For single molecule analysis, α-haemolysin was introduced at a low concentration (0.1 μg·mL^−1^) to allow stochastic incorporation of the α-haemolysin nanopore in lipid bilayers on aL-ALBiC^16^, *i.e*. most chambers had 0, 1, or 2 molecules of α-haemolysin as confirmed in our previous study^16^. The responses of individual chambers were far from homogeneous: some chambers showed clear fluorescence decay due to α-haemolysin activity, while others retained the original fluorescent signal (Fig. 3b,c), which supports the stochastic formation of α-haemolysin pores in aL-ALBiC. Figure 3b shows typical time courses of the exponential decay of Alexa 488 fluorescent signals after injection of α-haemolysin. The distribution of the decay rate constant exhibited three distinctive peaks (Fig. 4a). The intervals between peaks were essentially constant (Fig. 4a), which shows that each peak corresponds to the incorporation of 0, 1 or 2 α-haemolysin pores as previously reported^16^. The incorporation efficiency of α-haemolysin, calculated as the ratio of the total incorporated number of α-haemolysin pores to the total number of chambers, was the same (41.4%, Fig. 4b) among the three aL-ALBiCs even though the chamber volumes were different. This consistency is probably due to the uniform size of the lipid bilayers, which equalizes the incorporation efficiency of membrane proteins^12^. The rate constants for single transporter activity (*k*) determined from the median value of the second peak from the left in the distribution in Fig. 4a were 0.26 × 10^−3^, 1.1 × 10^−3^, and 4.0 × 10^−3^ s^−1^ for chambers with 471 nm, 116 nm, and 30 nm heights, respectively. Thus, the detection time for passive transport activity, *i.e*. the time constant of fluorescent decay, τ = 1/*k*, decreased in proportion to the chamber height (Fig. 4c), demonstrating that aL-ALBiC enables faster detection of single transport activity with stable transporter protein incorporation efficiency.

## Discussion

For efficient incorporation of transporters and rapid detection of membrane transport, the ratio of lipid bilayer area to chamber volume (S/V ratio) and the chamber volume itself are important: the S/V ratio should be as high as possible since a larger bilayer area increases the incorporation efficiency of membrane transporters, while a smaller chamber volume condenses the transported substrates^12^. The S/V ratio for aL-ALBiC (30,000 μm^2^/pL) is considerably higher than that of reported micro-systems^10^,^11^,^12^,^13^,^14^,^15^,^16^,^17^ and almost the same as 200-nm diameter liposomes, which is the most sensitive optical method for detection of membrane transport activity; however, liposomes are unsuitable for quantitative analysis due to heterogeneity in chamber volumes. As confirmed in the α-haemolysin assay (Fig. 3b), aL-ALBiC enables both prompt incorporation of transporters and rapid analysis of transporter activities due to the high S/V ratio, which is consistent with the above contention. Considering the highly quantitative nature of aL-ALBiC, this method, thus, achieves rapid and quantitative analysis of single transporter activities and has the potential to become a versatile method for membrane transporter studies.

The use of aL-ALBiC as a universal platform would enable detection and analysis of other transporters, receptors, and more complex bio-systems that work on a lipid bilayer. The shallow chamber height (30 nm) of aL-ALBiC can be combined with a total internal reflection (TIR) illumination system, which enables single-fluorophore detection by illuminating the very shallow area corresponding to the penetration depth of evanescent waves (100 nm). The TIRF system has been widely used for various biological assays and could increase the potential applications of aL-ALBiC, *e.g*. for direct observation of incorporated membrane proteins. The aL-ALBiC system could also be applied to encapsulate artificial cell-like systems under semi-physiological conditions due to its ultra-small volume, which would allow various kinds of cells to be fused into aL-ALBiC without any significant dilution of cell components. The regularly shaped structure and stability of aL-ALBiC may facilitate the quantitative analysis of such artificial cell systems. Additionally, the high-throughput feature of ALBiC-based transporter detection using more than 10,000 reactors may allow for its application in pharmacological screening of membrane transporters.

## Methods

### Microfabrication

Arrayed hydrophilic in hydrophobic structures were fabricated on a glass slide by conventional photolithography^16^,^17^,^18^,^19^. A hydrophobic carbon-fluorine polymer (CYTOP, Asahi-glass, Japan) was spin-coated on a SiO_2_ glass substrate and baked to evaporate the solvent. To prepare chambers with three different heights, we used 1.125%, 4.5%, and 9% (w/v) CYTOP. A positive photoresist (AZP4903, AZ Electronic Materials, Japan) was spin-coated on the CYTOP layer, and the patterning mask structure (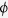, 3 μm; interval, 6 μm) was formed by UV-lithography. To expose the hydrophilic SiO_2_ glass surface, the resist-patterned substrate was etched with O_2_ plasma. Finally, the remaining photoresist was removed using acetone. We efficiently fabricated hydrophobic through-hole structures on a hydrophilic SiO_2_ glass substrate with a success rate of ~100%. The diameter of fabricated chambers was 3.0 ± 0.2 μm, and the heights were 30 ± 5 nm, 116 ± 13 nm, and 471 ± 42 nm, as measured by 3D laser scanning microscopy, atomic force microscopy, and transmission electron microscopy. To exchange solutions in the ALBiC systems, a flow cell was constructed from the fabricated nano-device, a spacer sheet, and a CYTOP-coated glass block with an access port for injection of solution.

### Imaging

The fluorescence intensities were recorded through a 60× objective lens with photomultiplier tubes (A1R, Nikon, Japan) using a field of view that encompassed ~100 micro-wells. Analysis was performed using NIS Elements software (Nikon).

### Passive transport assay for α-haemolysin

The chamber was filled with buffer A (0.1 mM HEPES, 20 mM NaCl, 2 mM MgCl_2_, pH adjusted with NaOH to 7.0) containing 10 μM Alexa 488 (A-10254, Life Technology, CA). Lipid bilayers were formed on the individual orifices of the microchamber array, then buffer A plus 0.1 μg/mL (400 pM) α-haemolysin (HT101, Toxin company, FL) was injected. Fluorescent images were recorded at 10, 20, or 40 s intervals for chambers with heights 30, 116, or 471 nm, respectively. All results reported in this work are the mean ± standard deviation of three or more measurements using three or more independent chips.

## Additional Information

**How to cite this article**: Soga, N. *et al*. Attolitre-sized lipid bilayer chamber array for rapid detection of single transporters. *Sci. Rep*. **5**, 11025; doi: 10.1038/srep11025 (2015).

## Supplementary Material

Supplementary Information
