# Supplementary material for: Attolitre-sized lipid bilayer chamber array for rapid detection of single transporters
Source: Sci Rep. 2015 Jun 8;5:11025. doi: 10.1038/srep11025 (PMC4458879; doi:10.1038/srep11025)
Supplement: Supplementary Information [file srep11025-s1.pdf]

Supplementary information

# Attolitre-sized lipid bilayer chamber array for rapid detection of single transporters

Naoki Soga, Rikiya Watanabe and Hiroyuki Noji

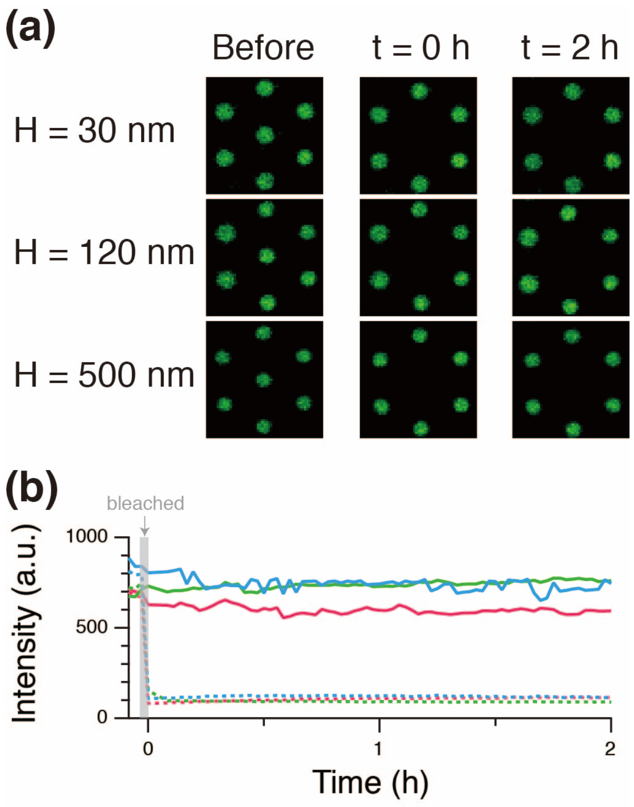


**Fig. S1** FRAP measurements of Alexa488 encapsulated in chambers (a) Fluorescence image of 10 μM Alexa488 in the chambers recorded before photobleaching (left), at 0 h (middle) and 2h (right) after photobleaching. (b) Time courses of the fluorescent intensity of the bleached chambers (dashed line) and their surrounding chambers (rigid line). The chambers were photobleached at t = 0 s (grey arrow). Color indicates the size of ALBiC: cyan; 3 fL, green; 800 aL, magenta; 200 aL. The traces are average over at least 20 chambers.

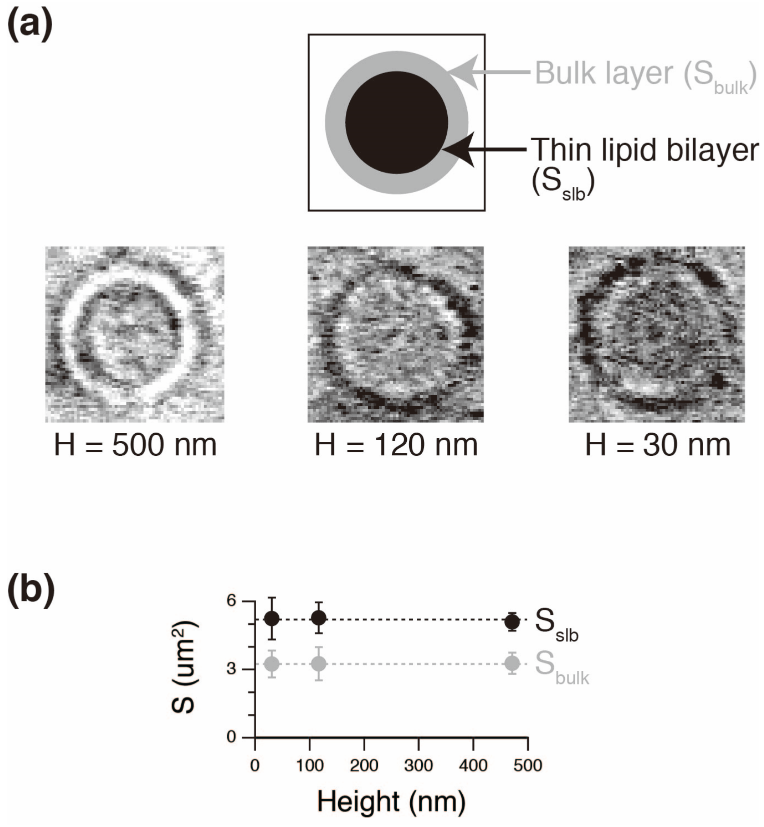


**Fig. S2** Bright field image of chambers after lipid bilayer formation (a) Bright field image of chambers with height of 471 nm (left), 116 nm (middle) and 30 nm (right). (b) Chamber-height dependence of the area of Plateau-Gibbs border ($S_{bulk}$) and the inner area surrounded by Plateau-Gibbs border ($S_{slb}$). The average areas were 5.2 μm$^2$ and 3.3 μm$^2$ for $S_{slb}$ and $S_{bulk}$, respectively.
